# Effect of thermal and chemical treatments used for SARS-COV-2 inactivation in the measurement of saliva analytes

**DOI:** 10.1038/s41598-022-13491-9

**Published:** 2022-06-08

**Authors:** Elsa Lamy, Camila P. Rubio, Laura Carreira, Fernando Capela e Silva, Silvia Martinez-Subiela, Fernando Tecles, Pia Lopez-Jornet, Jose J. Ceron, Asta Tvarijonaviciute

**Affiliations:** 1 MED-Mediterranean Institute for Agriculture, Environment and Development & CHANGE-Global Change and Sustainability Institute, University of Évora, Ap. 94, 7006-554 Évora, Portugal; 2 Interdisciplinary Laboratory of Clinical Analysis (Interlab-UMU), Veterinary School, Regional Campus of International Excellence Mare Nostrum, University of Murcia, Campus de Espinardo s/n, Espinardo, 30100 Murcia, Spain; 3 Department of Medical and Health Sciences, School of Health and Human Development, University of Évora, Évora, Portugal; 4 Interdisciplinary Laboratory of Clinical Analysis, Interlab-UMU, Regional Campus of International Excellence Mare Nostrum, University of Murcia, Espinardo, 30100 Murcia, Spain; 5 Faculty of Medicine and Odontology, Hospital Morales Meseguer, Clínica Odontológica, Marqués del los Vélez s/n, 30008 Murcia, Spain

**Keywords:** Diagnostic markers, Predictive markers, Biochemical assays, Proteomic analysis

## Abstract

The present study aims to assess the effects of thermal and chemical inactivating procedures, that can be used for SARS-CoV-2 inactivation, on different salivary analytes. SDS–Polyacrylamide Gel Electrophoresis (SDS-PAGE) protein profile and a panel of 25 specific biomarkers of oxidative status, stress, metabolism and tissue damage were evaluated in samples subjected to different treatments: thermal (65 °C or 92 °C) and chemical with detergents [sodium dodecyl sulphate (SDS), Triton X-100 or NP-40]. Salivary SDS-PAGE profile was most affected by heating at 92 °C, with three and two protein bands decreasing and increasing their expression levels, respectively. This treatment also affected the results of several enzymes, with some of them being also affected by heating at 65 °C and incubation with SDS. The use of Triton X-100 or NP-40 resulted in increased values of cortisol, triglycerides and glucose, not affecting the other tested biomarkers. The present results will help researchers and clinicians to select the best protocols to work in safe conditions with saliva, taking into account the target analyte planned to be measured.

## Introduction

Saliva became a fluid of interest due to its high potential for the study and evaluation of pathological and physiological conditions, together with its non-invasive nature, being increasingly used in a high number of different research fields, including human and animal oral and systemic health, oral food perception or stress and well-being^1^. Salivary proteome, obtained by the use of sodium dodecyl sulphate–polyacrylamide gel electrophoresis (SDS PAGE) or other techniques, has been used in clinical studies^2^, as well as in research areas related with ingestive behavior and food choices^3–5^. Moreover, many other salivary analytes, including enzymes, cortisol or uric acid, are also used to provide information about stress, oxidative status or tissue damage^5,6^. Despite the growing interest and potential of saliva analysis, saliva-based research in areas other than those related to COVID-19 diagnosis was limited during the COVID-19 pandemic. This situation is because saliva contains the coronavirus SARS-CoV-2, the etiologic agent of the disease^7–11^, which raises significant concerns for guarantee the researcher's security when saliva is used. Even after two years after the starting of pandemics, and with vaccines available, health authorities still recommend the use of masks and hygiene measures to avoid contaminations through oral and nasal fluids. As such, a high pressure is still put on saliva manipulations. Similarly, saliva may be a source of other virus contaminations. In this sense, the use of protocols for virus inactivation before any saliva analysis can be a way to circumvent the reported limitations.

The need of working with biological samples in a safe way resulted in the search for methods able to inactivate SARS-CoV-2^12^. Therefore, different protocols have been tested for specific inactivation of this virus, including physical and chemical inactivation methods. Physical inactivation can be achieved by heat or ultra-violet (UV) radiation, whereas chemical inactivation is based on different chemicals (e.g. detergents and trizol) that act at different levels^12,13^. The inactivation due to thermal treatment acts at the level of the virus's protein coat, destroying the structure of the proteins. Total inactivation of the virus in biological samples was achieved at temperatures of 80 °C, for 1 h^13^. It was recently demonstrated that heating at 92 °C, for 15 min, is more effective than 56 °C for 30 min or even than 60 °C for 60 min, to achieve 6-log reductions^12^. Concerning UV light exposure, the application of UV energy > 0.04 J/cm^2^ was observed to inactivate SARS-CoV-2^13^ and described with other coronaviruses^14^.

Besides physical methods, chemical procedures consisting of ethanol, peracetic acid, or chlorine-containing disinfectants inactivate SARS-CoV-2^15^. Also, the use of non-ionic detergents, such as NP-40 or Triton X-100, and ionic detergents such as SDS, were proven to be effective against this virus^13^.

It is known that the temperature and general conditions used in the storage and processing of saliva samples can affect the results of different analytes, including relevant salivary enzymes, such as amylase, cholinesterase, lipase, among others^16^. At the same time, different chemicals can have diverse effects and interferences in the measurement of different molecules. Therefore, the choice of the most suitable inactivation protocol should be made by selecting those producing the minor effects and analytical inaccuracies in the analytes of interest.

The present study aimed to access the possible effect of physical/thermal (65 °C and 92 °C) and chemical (SDS, NP40 and triton) treatments, which are reported as effective for SARS-CoV-2 inactivation, on the salivary SDS-PAGE protein profile and biochemistry analytes that are usually determined in saliva. The salivary profile of analytes evaluated in this study consisted of 25 analytes and included biomarkers related to oxidative status such as Cupric Reducing Antioxidant Capacity (CUPRAC), ferric reducing ability of saliva (FRAS), Trolox equivalent antioxidant capacity (TEAC), uric acid, catalase (CAT), total esterase activity (TEA), the advanced oxidation protein products (AOPP) and hydrogen peroxide (H_2_O_2_). In addition, it included biomarkers of stress [salivary alpha-amylase (sAA), cortisol, immunoglobulin A (IgA)] and biomarkers related to metabolism and tissue damage [total proteins, aspartate transaminase (AST), alanine aminotransferase (ALT), creatinine kinase (CK), lactate dehydrogenase (LDH), lactate, urea, creatinine, total cholesterol, triglycerides, cholinesterase (ChE), glucose, calcium (Ca), phosphorus (P)].

## Materials and methods

### Samples

A total of 18 saliva samples were used in this study. For SDS-PAGE analysis, saliva samples from eight donors (4 women and 4 men) between 20 and 25 years were used. Whole saliva samples were collected by the passive drool method following previously reported methodologies^3^. In brief, the participants were asked to not eat for at least an hour before and to rinse the mouth thoroughly 5 min before saliva sample collection to avoid possible contamination of the sample. Obtained saliva samples (approx. 5 mL) were slightly vortexed and centrifuged (3000×*g* for 10 min at 4 °C) and the supernatant was aliquoted. Samples for proteomics were stored at − 30 °C until analysis (no longer than two months).

For biochemistry analysis, saliva samples were obtained from ten subjects (5 women and 5 men) between 25 and 35 years, as described above, and treated and analyzed in fresh.

In the present study saliva samples from subjects testing positive to SARS-CoV-2 were not included, since our aim was to assess the specific effect of the heat- or chemical methods in salivary molecules analysis and this effect is not expected to be affected by the presence or absence of virus.

All procedures were in accordance with the Declaration of Helsinki given by World Medical Association, assuring the inexistence of invasiveness, and included in a project approved by the Ethical Committee of the University of Evora (GD/6543/2021). An informed consent was obtained from all the participants, after receiving information about the objective of the study and the type of data to be obtained by the collected saliva samples.

### Inactivation protocols

Six aliquots of each sample were prepared and simultaneously submitted to following treatment protocols: TT1—control (no inactivation treatment); TT2—heated at 65ºC for 30 min; TT3—heated at 92ºC for 15 min; TT4—mixed with SDS (10% w/v) to a final concentration of 0.5% SDS followed by immediate incubation at RT for 30 min; TT5—treated with NP-40 (10% v/v) to a final concentration of 0.5% of NP-40 followed by immediate incubation for 30 min; TT6—treated with Triton X-100 (10% v/v) to a final concentration of 0,5% of Triton X-100 followed by immediate incubation, at room temperature for 30 min. All these inactivation procedures were done in duplicate.

### SDS-PAGE

Due to the possible different effects, of each inactivation protocol, in the results obtained from total protein assays^17^, the total protein concentration of controls (TT1) was used for calculation of the volume to be used in SDS-PAGE runs. Each saliva sample was run in duplicate and, for each, a volume corresponding to 7.5 µg total protein was mixed with sample buffer and run on each lane of 14% polyacrylamide mini-gels (Protean xi, Bio-Rad, CA, USA) using a Laemmli buffer system, as described elsewhere^2^. In each gel, one of the lanes was used for molecular mass standard (Bio-Rad Precision Plus Protein Dual Colour 161-0394) running. The electrophoretic run was performed at a constant voltage of 140 V until the front dye reached the gel's end. Gels were fixed for 1 h in 40% methanol/10% acetic acid, followed by staining for 1 h with Coomassie Brilliant Blue (CBB) R-250 and destained in several washes of 10% acetic acid. Gel images were acquired using a scanning Molecular Dynamics densitometer with internal calibration and LabScan software (GE Healthcare), and images were analyzed using GelAnalyzer software (GelAnalyzer 2010a by Istvan Lazar, https://www.gelanalyzer.com) for the volume percentage of each protein band.

### Biochemical analytes and antioxidant quantification

Saliva total protein, alpha-amylase (sAA), Cholinesterase (ChE), total esterase activity (TEA), creatinine kinase (CK), aspartase transaminase (AST), lactate dehydrogenase (LDH), lactate, ferric reducing ability of saliva (FRAS), the Trolox equivalent antioxidant capacity (TEAC), cupric reducing antioxidant capacity (CUPRAC), uric acid, catalase (CAT), the advanced oxidation protein products (AOPP), hydrogen peroxide (H_2_O_2_), glucose, triglycerides, creatinine, calcium (Ca), total cholesterol, phosphorus (P), alanine aminotransferase (ALT) and urea were determined using previously validated commercial kits or in home-made assays for use in saliva samples^5,16^. An automated biochemistry analyzer was used for all the measurements (Olympus AU600, Olympus Diagnostica GmbH, Freiburg, Germany).

Total IgA was evaluated with a commercial ELISA kit (Bethyl, Montgomery, TX, USA) previously validated for use with human saliva samples^18^. Cortisol was analyzed with an immunoassay system (Immulite, Siemens Healthcare Diagnostics. Deerfield, IL)^19^.

### Statistical analysis

All data were tested for normal distribution using Shapiro–Wilk. Since most parameters were not normally distributed and due to the low number of samples (N = 8, for SDS-PAGE analysis and N = 10 for biochemical parameters), non-parametric tests were used. Friedman test was used to compare each salivary parameter among the six protocols used, followed by Dunn's pairwise post hoc tests with Bonferroni correction for multiple testing. Due to the independence of the inactivation treatments, among each other, in cases where Friedman resulted in non-significant variations among the protocols and, consequently Dunn's pairwise post hoc tests were not performed, inactivation treatments were compared with control using Wilcoxon test. Statistical analysis was performed using SPSS v. 24 software, with significance level set at 5%.

## Results

### SDS-PAGE analysis

**Figure 1:**
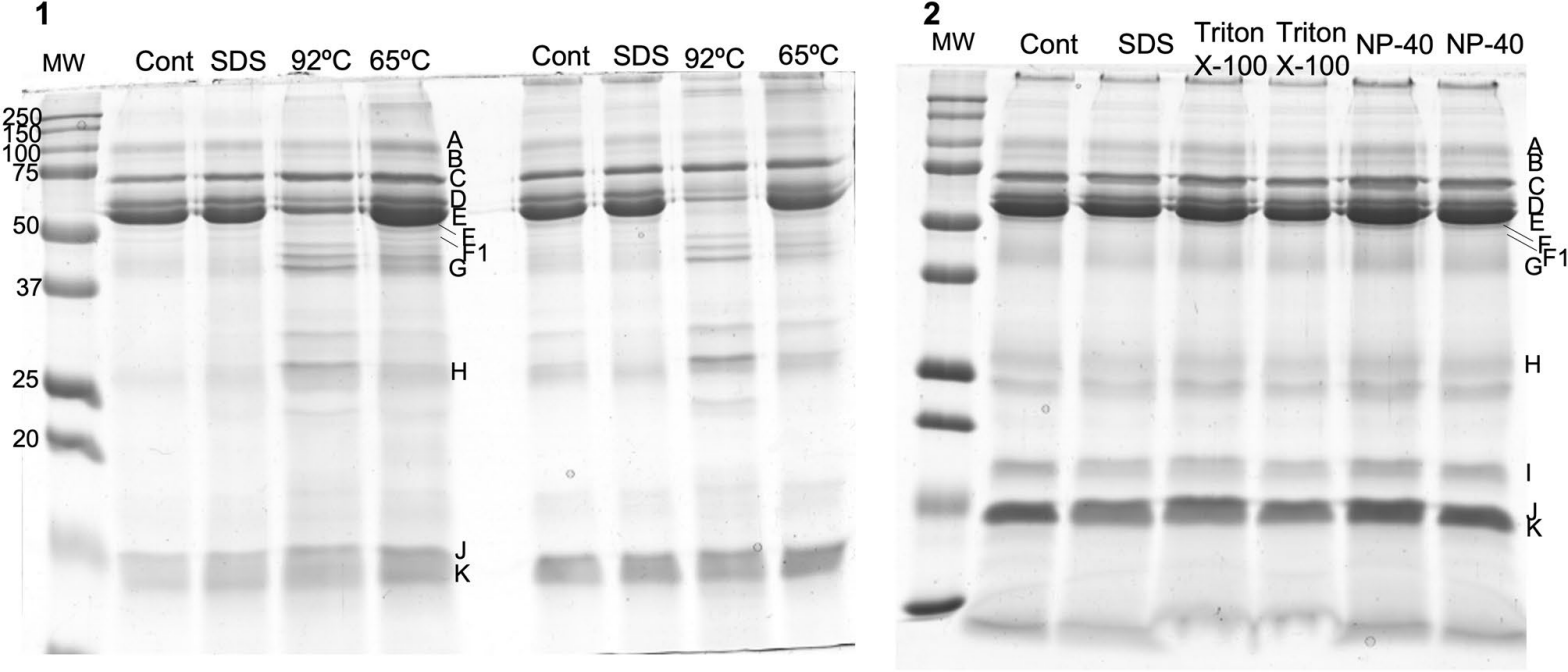
Example of SDS-PAGE profiles of the same individual’s saliva sample subjected to the different treatments: (**1**) Control, SDS, heat treatment at 92 °C; heat treatment at 65 °C; (**2**) control, SDS, Triton X-100; NP-40; (letters represent the protein bands considered for analysis, which were previously identified as containing: A—IgG polymeric receptor; B-—transferrin; C—albumin; D, E and F—salivary amylase; F1—not identified; G—Zinc-α2-glycoprotein + Carbonic anhydrase VI; H—immunoglobulin light chains; I—prolactin induced protein; J and K—cystatins; MW—molecular mass standard).

Twelve protein bands were observed to be well resolved in all gels and able to be compared among treatments (Fig. 1).

Concerning SDS-PAGE results, thermic treatments produced major variations in the protein profile, particularly heating at 92 °C for 15 min (Supplementary Table 1). Lower expression levels of the bands A, D and E and higher expression levels of the bands H and J were observed when this inactivation protocol was used. According to previous identifications^3^, bands D and E contain salivary amylase, in glycosylated and native form, respectively, whereas bands H and J contain immunoglobulin chains and type-S cystatins, respectively.

No significant changes in the SDS-PAGE profiles due to the use of detergents, were observed when Friedman test was used for analysis. However, comparisons of each treatment directly with control, using Wilcoxon test, showed that the use of both NP-40 and Triton X-100 resulted in a decrease in the expression levels of band E, which corresponds to the native form of amylase. Inactivation using the SDS treatment did not result in significant changes in any of the protein band of the SDS-PAGE profiles.

### Biochemical analytes and antioxidant quantification

When effects of different inactivation treatments were assessed on 25 individual analytes in saliva, TT2 (62 °C, 30 min) had a statistically significant effect on values of seven analytes, TT3 (92 °C, 15 min) on ten analytes, TT4 (SDS) on 13 analytes, TT5 (NP40) on five analytes and TT6 (Triton X–100) on seven analytes (Tables 1, 2 and 3).

**Table 1:** Effect of inactivation protocols in salivary total protein and oxidative status biomarkers [median (25–75 percentile)].

Analyte	TT1	TT2	TT3	TT4	TT5	TT6	P
Total protein, g/L	49.76 (33.425—115.05)	59.88 (42.6675—83.865)	62.50 (40.78—79.4275)	60.11 (41.2175—84.32)	56.26 (43.2775—112.695)	70.05 (47.5625—114.25)	0.063
CUPRAC, mmol/L	0.256 (0.213—0.278)	0.257 (0.225—0.281)	0.259 (0.221—0.281)	0.257 (0.218—0.275)	0.263 (0.221—0.276)	0.274* (0.241—0.295)	**0.002**
FRAS, mmol/L	0.429 (0.356—0.519)	0.421 (0.368—0.514)	0.421 (0.359—0.513)	0.441 (0.358—0.500)	0.440 (0.364—0.515)	0.444 (0.360—0.519)	0.501
TEACH, mmol/L	0.259 (0.206—0.287)	0.267* (0.220—0.298)	0.256 (0.213—0.293)	0.252 (0.213—0.282)	0.253 (0.210—0.288)	0.252 (0.206—0.283)	** < 0.001**
Uric acid, mmol/L	0.201 (0.101–0.234)	0.203* (0.104–0.234)	0.204* (0.110–0.244)	0.200 (0.101–0.230)	0.200 (0.102–0.240)	0.202 (0.103–0.234)	** < 0.001**
CAT, IU/L	298 (210–331)	345 (167–385)	0* (0–0)	306 (79–355)	0.2795 (204–371)	287 (0.197–380)	** < 0.001**
TEA, IU/L	30.0 (23.875–55.8)	16.55* (9.725–29.775)	15.3* (6.5–25.35)	15.4* (6.4–27.5)	20.7 (16.975–42.675)	21.55 (15.6–40.225)	** < 0.001**
AOPP, µmol/L	163.4 (106.625–249.425)	173.6 (105.05–230.15)	191.3 (99.975–250.45)	111.65* (73.15–146.6)	91.2* (63.375–170.525)	87.55* (61.25–152.775)	** < 0.001**
H_2_O_2_, µmol/L	7.15 (4.7–10.05)	8.4 (4.275–11.575)	9.3 (4.775–12.75)	8.3 (5.25–12.425)	4.95 (3–8.9)	3.7* (1.725–7.95)	** < 0.001**

Significant values are in bold.

*P < 0.05. Control (TT1); 65 °C 30 min (TT2); 92 °C 15 min (TT3); SDS (TT4); NP40 (TT5) and triton (TT6).

**Table 2:** Effect of inactivation protocols in salivary stress biomarkers [median (25–75 percentile)].

Analyte	TT1	TT2	TT3	TT4	TT5	TT6	P
SAA, IU/L	50,141.8 (26,220.5–115,394.9)	887.3* (539.1–4860.6)	0* (0–14.3)	51,992.6 (1459.6–99,253.5)	66,760.4 (28,214.4–186,109.3)	67,523.6 (28,844.0–188,647.0)	** < 0.001**
Cortisol, nmol/L	12.97 (9.66–15.73)	11.59 (8.83–16.55)	11.86 (8.55–14.90)	139.04* 127.5–191.5)	119.73* (112.3–138.5)	142.4* (131.6–153.9)	** < 0.001**
IgA, g/L	0.169 (0.106–0.369)	0.141 (0.068–0.233)	0.016* (0.010–0.081)	0.025* (0.009–0.127)	0.170 (0.118–0.273)	0.172 (0.116–0.287)	** < 0.001**

Significant values are in bold.

*P < 0.05. Control (TT1); 65 °C 30 min (TT2); 92 °C 15 min (TT3); SDS (TT4); NP40 (TT5) and triton (TT6).

**Table 3:** Effect of inactivation protocols in salivary biomarkers of metabolic status, kidney function and tissue damage [median (25–75 percentile)].

Analyte	TT1	TT2	TT3	TT4	TT5	TT6	P
AST, IU/L	19.00 (16.5–20.95)	7.9* (4.65–10.08)	0.35* (0.15–0.85)	0.75* (0.15–1.7)	18.65 (13.3–20.95)	19.45 (14.1–20.625)	** < 0.001**
ALT, IU/L	5.3 (3.825–7)	1.65* (1.075–2.25)	1.6* (1.375–2.075)	1.55* (1.2–2.2)	4.45 (2.925–6.1)	4.05 (2.95–6.2)	** < 0.001**
CK, IU/L	19.75 (12.375–29.5)	0.25* (0–2.075)	0.3* (0–1.05)	0.7* (0–3)	15.95 (12.325–20.675)	17.6 (12.175–21.5)	** < 0.001**
LDH, IU/L	293.3 (220.625–420.175)	4.45* (2.75–5.8)	1.8* (0.525–3.375)	1.5* (0.45–4.05)	283.6 (214.2–406.325)	282.95 (216.375–417.8)	** < 0.001**
Urea, mmol/L	9.98 (8.60–10.75)	10.0 (8.59–11.02)	10.3 (8.76–11.21)	9.10* (7.80–9.85)	9.69 (8.31–10.77)	9.73 (8.59–11.08)	** < 0.001**
Creatinine, µmol/L	7.96 (5.08–11.05)	7.96 (5.08–11.27)	6.19 (1.77–11.71)	7.51 (5.75–10.17)	7.51 (5.30–11.27)	7.51 (6.19–9.50)	0.382
Total cholesterol, mmol/L	6.1 × 10^–3^ (9.8 × 10^–4^–8.5 × 10^–3^)	1.19 × 10^–2^ (5.18 × 10^–3^–1.67 × 10^–2^)	1.26 × 10^–2^ (1.09 × 10^–2^–1.42 × 10^–2^)	8.29 × 10^–3^ (1.17 × 10-3–1.84 × 10^–2^)	8.03 × 10^–3^ (0–1.02 × 10^–2^)	1.18 × 10^–2^ (2.85 × 10^–3^–1.37 × 10^–2^)	**0.0417**
Triglycerides, mmol/L	0.031 (0.027–0.055)	0.036 (0.025–0.057)	0.034 (0.017–0.062)	0.154* (0.120–0.188)	0.105* (0.091–0.126)	0.104* (0.093–0.117)	** < 0.001**
Glucose, mmol/L	0.014 (4.44 × 10^–3^–5,6 × 10^–2^)	8.32 × 10^–3^ (3.89 × 10^–3^–6.32 × 10^–2^)	3.33 × 10^–3^ (5,56 × 10^–4^–5,49 × 10^–2^)	2.00 × 10-2* (5.56 × 10^**–4**^**–9.88 × 10**^**–2**^**)**	**2.83 × 10**^–2^* (7.22 × 10^**–3**^**–0.110)**	**2.44 × 10**^–2^* (7.22 × 10^**–3**^**–0.109)**	** < 0.001**
CA, mmol/L	1.28 (1.05–1.39)	1.05 (0.85–1.29)	1.04 (0.90–1.29)	1.21 (1.03–1.44)	1.24 (1.03–1.38)	1.24 (1.03–1.42)	0.114
P, mmol/L	4.70 (4.08–5.49)	4.72 (4.27–5.42)	4.74 (4.23–5.56)	4.51* (3.79–5.25)	4.49 (4.17–5.37)	4.59 (4.17–5.49)	**0.001**
Lactate, µmol/L	1.755 (0.905–2.33)	1.805 (0.985–2.3275)	1.8 (0.94–2.295)	1.765 (0.3425–2.3125)	1.705* (0.8175–2.113)	1.745* (0.855–2.178)	** < 0.001**
ChE, IU/L	4.1 (2.3–8.45)	3.1 (1.3–5.4)	2.3* (0.8–3.6)	2.8* (1.6–5.2)	3.9 (1.3–6.0)	4.2 (1.4–5.8)	** < 0.001**

Significant values are in bold.

*P < 0.05. Control (TT1); 65 °C 30 min (TT2); 92 °C 15 min (TT3); SDS (TT4); NP40 (TT5) and triton (TT6).

In the biomarkers of oxidative status (CUPRAC, FRAS, TEAC, uric acid, CAT, TEA, AOPP, H_2_O_2_), the treatment with 92 °C for 15 min affected the higher number of biomarkers and produced changes in their values that were of higher magnitude compared to the controls. Whereas NP40 only produced significant changes in AOPP (Table 1).

In the biomarkers of stress (cortisol, sAA and IgA), the heat treatments produced a major decrease in sAA and IgA. However, in this last case, only 92ºC treatment had effect. In the case of cortisol, thermal treatments did not have a significant effect. Whereas the use of detergents (SDS, Triton X-100 and NP-40) did not produce significant changes in sAA, it resulted in a marked increase in cortisol values (Table 2).

For the biomarkers related to the metabolic status, kidney function and tissue damage, the heat treatments produced significant decreases in all enzymes (AST, ALT, CK and LDH), and detergents produced a significant increase in triglycerides and glucose (Table 3).

## Discussion

The current situation of COVID-19 pandemic and the easy transmission of SARS-CoV-2, through saliva, resulted in the need for particular care for the laboratory handling of this fluid. Procedures that are already known to inactivate this virus should be used before any saliva analysis. However, this raises the concern about the effect that these inactivation treatments may have on the analytes that can be measured in saliva and that are widely used for information about stress, oxidative status and tissue damage. The present study compared the effect of different known inactivation protocols in the quantification of different salivary analytes.

One of our main observations was that none of the treatments allows the maintenance of all salivary analytes in the same levels as in untreated samples: some inactivation protocols affected the levels of some salivary parameters, whereas other protocols affected different ones. As such, the protocol of choice must consider the objective of the study and the type of molecules to be evaluated.

Heat treatment at 92 °C, for 15 min, was the treatment that affected a higher number of protein bands in SDS-PAGE profiles. This was not a major surprise, since the effect of heat in protein mixtures and proteins structures can be variable, but it usually results in denaturation and/or aggregation^20,21^. In the case of heat-aggregation, is has been described that bovine serum albumin (BSA) started to be affected at above 60 °C, whereas for myosin 50 °C is enough to induce the aggregation process^22^. Also different heating temperatures resulted in different effects in SDS-PAGE profiles of some food proteins^22^. This is in line with our saliva results, where heat treatment at 65 °C and 92 °C resulted in different effects on the protein bands. Concerning detergents, SDS had no effects in SDS-PAGE profiles at the concentration used, which was expected since this is the ionic detergent used in sample preparation when proteins are separated using this electrophoretic technique. The non-ionic detergents tested (NP-40 and Triton X-100) resulted in reduced expression levels of the protein band containing the native form of amylase, comparatively to control. The reason why this happened specifically to this band needs to be further elucidated.

For the biochemical assays, heat treatment and SDS produced a significant decrease in most of the enzymes evaluated in our study (TEA, ChE, AST, ALT, CK and LDH). The inhibitory effect of SDS on enzymes activity has been described previously^23^. In case of SDS being needed, it is possible to revert the inhibitory effect for some enzymes^24^, but this should be explored in saliva. In the case of sAA heat treatments inhibit it, but SDS kept its activity. Whereas both heating treatments decreased the enzymatic activity of the protein, losses in the expression levels of the SDS-PAGE bands containing amylase were only observed when heating at 92 °C. This means that temperatures of 65 °C seems not to produce structural changes in sAA but can affect its activity. This is particularly relevant since amylase is one of the salivary analytes more frequently analyzed^4,25–27^.

Triton X-100 and NP-40 did not affect enzymes but produced an increase in cortisol, triglycerides and glucose. This could be related to their effect on the liberation of cell membrane proteins and components in general or due to direct interferences with the methods of measurements. The lack of effect in enzymes would be in line with a previous recent report in which these two chemicals were used in the measurement of the enzyme adenosin deaminase in saliva, not affecting the quantification^28^. Regarding biomarkers of oxidative stress, these two detergents produced a decrease in AOPP, which should be taken into consideration when this analyte is up to be determined. In the particular cases of lactate and CUPRAC, the detergents produced changes with statistical significance, although these variations were minor and probably would not have a major effect on the result interpretation. Nevertheless, they need to be considered.

One limitation of the present study was the lack of testing these treatments in saliva samples containing SARS-CoV-2. As such, it is not possible to exclude modifications in SDS PAGE profiles, due to the proteinaceous part of the virus, for example. For enzymatic activities and the other parameters tested, an effect of virus presence on the results are not expected. Nevertheless, it can be of interest to explore this situation in further studies.

In conclusion, among the different inactivation treatments, for saliva SDS-PAGE analysis it is advisable to use SDS for virus inactivation, while for biochemical assays, Triton X-100 and NP-40 can be used in the determination of most of the analytes evaluated in the present study, with the exception of triglycerides, cortisol, glucose and AOPP. For these last, heating treatments can be recommended. It is expected that these results could help to choose the better sample treatment for SARS-CoV-2 inactivation according to the analyte that is going to be measured in saliva.

The results of the present study may be of interest for different situations, where saliva samples need to be thermally or chemically treated for different purposes.

## Supplementary Information

Supplementary Table 1.

## References

[1] Tvarijonaviciute, A., Martinez-Subiela, S., Lopez-Jornet, P. & Lamy, E. *Saliva in Health and Disease the Present and Future of a Unique Sample for Diagnosis*. 10.1007/978-3-030-37681-9 (Springer Wiley, 2020).

[2] Lamy E (2015). Changes in the salivary protein profile of morbidly obese women either previously subjected to bariatric surgery or not. J. Physiol. Biochem..

[3] Carreira L (2020). Changes in salivary proteome in response to bread odour. Nutrients.

[4] Rodrigues L (2019). Comparison of salivary proteome of children with different sensitivities for bitter and sweet tastes: association with body mass index. Int. J. Obes..

[5] Tvarijonaviciute A (2020). Saliva as a non-invasive tool for assessment of metabolic and inflammatory biomarkers in children. Clin. Nutr..

[6] Lopez-Jornet P (2020). Salivary biomarkers and their correlation with pain and stress in patients with burning mouth syndrome. J. Clin. Med..

[7] Azzi L (2020). Saliva is a reliable tool to detect SARS-CoV-2. J. Infect..

[8] Ceron JJ (2020). Use of saliva for diagnosis and monitoring the SARS-CoV-2: A general perspective. J. Clin. Med..

[9] Sabino-Silva R, Jardim ACG, Siqueira WL (2020). Coronavirus COVID-19 impacts to dentistry and potential salivary diagnosis. Clin. Oral Invest..

[10] Zheng S (2020). Saliva as a diagnostic specimen for SARS-CoV-2 by a PCR-based assay: A diagnostic validity study. SSRN Electron. J..

[11] Lalli M (2020). Rapid and extraction-free detection of SARS-CoV-2 from saliva with colorimetric LAMP. medRxiv Preprint Serv. Health Sci..

[12] Pastorino B, Touret F, Gilles M, de Lamballerie X, Charrel RN (2020). Heat inactivation of different types of SARS-CoV-2 samples: What protocols for biosafety, molecular detection and serological diagnostics?. Viruses.

[13] Patterson EI (2020). Methods of inactivation of SARS-CoV-2 for downstream biological assays. J. Infect. Dis..

[14] Darnell MER, Subbarao K, Feinstone SM, Taylor DR (2004). Inactivation of the coronavirus that induces severe acute respiratory syndrome, SARS-CoV. J. Virol. Methods.

[15] Zhou P (2020). A pneumonia outbreak associated with a new coronavirus of probable bat origin. Nature.

[16] Barranco T (2019). Changes of salivary biomarkers under different storage conditions: Effects of temperature and length of storage. Biochem. Med..

[17] Friedenauer S, Berlet HH (1989). Sensitivity and variability of the Bradford protein assay in the presence of detergents. Anal. Biochem..

[18] Lopez-Jornet P (2016). Oral lichen planus: Salival biomarkers cortisol, immunoglobulin A, adiponectin. J. Oral Pathol. Med..

[19] Tecles F (2014). Assessment of stress associated with an oral public speech in veterinary students by salivary biomarkers. J. Vet. Med. Educ..

[20] Lyubarev AE, Kurganov BI, Orlov VN, Zhou HM (1999). Two-state irreversible thermal denaturation of muscle creatine kinase. Biophys. Chem..

[21] Bin YY (2003). Two-dimensional infrared correlation spectroscopy study of sequential events in the heat-induced unfolding and aggregation process of myoglobin. Biophys. J..

[22] Kajak-Siemaszko K (2011). Characterization of protein aggregates following a heating and freezing process. Food Res. Int..

[23] Vincenzini MT, Favilli F, Stio M, Vanni P, Treves C (1985). Detergents as selective inhibitors and inactivators of enzymes. Physiol. Chem. Phys. Med. NMR.

[24] Weber K, Kuter DJ (1971). Reversible denaturation of enzymes by sodium dodecyl sulfate. J. Biol. Chem..

[25] Nater UM, Rohleder N (2009). Salivary alpha-amylase as a non-invasive biomarker for the sympathetic nervous system: current state of research. Psychoneuroendocrinology.

[26] Rodrigues L (2017). Salivary proteome and glucose levels are related with sweet taste sensitivity in young adults. Food Nutr. Res..

[27] Nater UM, Rohleder N (2009). Salivary alpha-amylase as a non-invasive biomarker for the sympathetic nervous system: Current state of research. Psychoneuroendocrinology.

[28] Franco-Martínez L (2021). Analytical validation of an automated assay for the measurement of adenosine deaminase (ADA) and its isoenzymes in saliva and a pilot evaluation of their changes in patients with SARS-CoV-2 infection. Clin. Chem. Lab. Med..

