# Supplementary material for: Effect of thermal and chemical treatments used for SARS-COV-2 inactivation in the measurement of saliva analytes
Source: Sci Rep. 2022 Jun 8;12:9434. doi: 10.1038/s41598-022-13491-9 (PMC9174913; doi:10.1038/s41598-022-13491-9)
Supplement: Supplementary file 1 — Supplementary Table 1. [file 41598_2022_13491_MOESM1_ESM.docx]

**Supplementary Table 1.** Median (25 – 75 percentile) data of SDS PAGE bands (%Vol) in non-treated samples (TT1) and in samples treated with 65 °C 30 min (TT2), 92 °C 15 min (TT3), SDS (TT4), NP40 (TT5) and triton (TT6).

| band  (% Vol) | TT1 | TT2 | TT3 | TT4 | TT5 | TT6 | P | Protein ID (obtained previously[3]) |
| --- | --- | --- | --- | --- | --- | --- | --- | --- |
| A | 5.48  4.73 – 6.24 | 5.36  4.79 – 6.25 | **4.40^#^**  **4.03 – 4.77** | 4.64  4.49 – 5.40 | 5.09  4.62 – 6.46 | 5.36  4.37 – 6.38 | 0.077 | Ig polymeric receptor |
| B | 1.77  .60 – 2.28 | 1.22  .37 – 2.49 | 1.34  .37 – 2.13 | 1.60  .63 – 2.22 | 1.38  .37 – 2.20 | 1.19  .37 – 2.00 | 0.637 | *n.i* |
| C | 10.74  9.42 – 11.99 | 9.35  6.46 – 10.13 | 10.06  9.47 – 12.97 | 9.96  8.96 – 11.56 | 10.79  9.20 – 11.63 | 10.51  8.62 – 13.27 | 0.382 | Albumin |
| D | 11.62  10.61 – 13.23 | 11.59  8.92 – 13.25 | **7.66^#^**  **6.59 – 11.18** | 11.60  8.92 – 13.25 | 10.87  9.90 – 11.32 | 8.97  7.69 – 11.70 | 0.064 | Salivary α-amylase |
| E | 21.87  18.67 – 23.41 | 19.52  16.95 – 23.31 | **11.40^#^**  **6.86 – 20.01** | 20.44  15.81 – 23.37 | **17.92^#^**  **16.11 – 18.86** | **16.82^#^**  **15.03 – 19.37** | **0.011*** | Salivary α-amylase |
| F | 3.21  2.22 – 3.97 | 2.98  .87 – 3.31 | 1.13  .37 – 2.84 | 2.89  2.43 – 3.31 | 3.18  2.98 – 3.77 | 3.20  2.66 – 3.58 | 0.438 | Salivary α-amylase |
| F1 | 2.12  1.84 – 2.48 | 2.12  .37 – 3.92 | 1.89  .37 – 3.38 | 2.46  2.08 – 3.45 | 2.09  1.47 – 2.51 | 2.23  1.97 – 2.71 | 0.674 | *n.i.* |
| G | 8.56  7.55 – 9.37 | 9.70  8.01 – 10.20 | 9.94  6.61 – 11.13 | 7.31  6.49 – 8.64 | 9.22  8.40 – 10.04 | 9.01  8.47 – 10.85 | 0.152 | Zinc α-2 glycoprotein + Carbonic Anhydrase VI |
| H | 7.37  6.65 – 10.49 | 7.69  6.96 – 10.84 | **12.81***  **6.83 – 14.52** | 8.33  7.40 – 11.62 | 9.22  7.63 – 12.42 | 9.24  8.20 – 12.22 | **0.036*** | Immunoglobulin kappa constant |
| I | 5.83  4.13 – 6.39 | **3.84***  **1.17 – 4.78** | 5.24  2.91 – 6.09 | 5.98  4.78 – 6.23 | 5.59  5.04 – 6.74 | 6.04  4.91 – 7.45 | **0.001*** | Prolactin-inducible protein |
| J | 9.04  5.82 – 11.50 | 10.31  6.73 – 15.07 | **12.61***  **11.32 – 14.54** | 9.51  7.89 – 11.41 | 9.83  6.40 – 10.31 | 8.87  5.78 – 10.30 | **0.001*** | Cystatin-SN |
| K | 10.71  6.23 – 15.88 | 11.99  6.31 – 14. 00 | 12.92^1^  12.37 – 15.02 | 11.47  8.99 – 14.30 | 9.86^1^  7.20 – 12.74 | 11.12^1^  7.82 – 12.26 | **0.009*** | Cystatin-S |

* Significantly different from control, using Friedman test followed by Dunn’s pairwise post hoc tests with Bonferroni correction for multiple testing; # Statistically significant using Wilcoxon for direct comparison with control; ^1^ Control did not present significant differences to treatments, but differences were observed between treatment 3 and the treatments 5 and 6.
